# Harnessing traditional healers role in strengthening Africa’s public health response: A case study of Mpox outbreak

**DOI:** 10.1371/journal.pntd.0013655

**Published:** 2025-10-29

**Authors:** Augustus Osborne, Umaru Sesay, Gebrekrstos Negash Gebru, Peter Bai James

**Affiliations:** 1 Institute for Development, Freetown, Sierra Leone; 2 Sierra Leone Field Epidemiology Training Program, National Public Health Agency, Freetown, Sierra Leone; 3 African Field Epidemiology Network, Freetown, Sierra Leone; 4 Africa Centres for Disease Control and Prevention, Addis Ababa, Ethiopia; 5 Faculty of Health, National Centre for Naturopathic Medicine, Southern Cross University, Lismore, Australia; 6 Faculty of Pharmaceutical Sciences, College of Medicine and Allied Health Sciences, University of Sierra Leone, Freetown, Sierra Leone; Public Health Agency of Canada, CANADA

## Abstract

Mpox, a zoonotic viral disease endemic in Central and West Africa, has posed major public health challenges, particularly following the 2022 outbreak. The World Health Organization declared it a public health emergency of international concern in July 2022. In Africa, the ongoing outbreak has exacerbated existing healthcare system weaknesses, further strained by civil conflict, poverty, and recurring diseases like Marburg, Lassa fever, and Ebola. Between January 1, 2022, and September 28, 2025, a total of 54,906 Mpox cases and 239 deaths were recorded from 30 WHO member states in Africa. Traditional healers, often the first contact for approximately 58% of the African population for treatment and management of illness, play a crucial role in the treatment of febrile illnesses like malaria, measles, and Mpox. Without effective engagement and regulation of informal health providers, their activities can contribute to delayed access to biomedical care, spread of misinformation, and non-adherence to infection prevention and control measures. By following medical pluralism/healthcare seeking behaviour framework to guide our argument, this viewpoint investigated the role of traditional healers in Mpox outbreaks and proposes strategies for effective collaboration in future outbreaks. Despite challenges like delayed case detection and misinformation, integrating traditional healers into public health responses offers opportunities to strengthen outbreak management. Strategies include training traditional healers in early symptom identification and referral protocols, engaging them in public health campaigns, and establishing clear referral systems to formal healthcare facilities. Providing infection prevention and control tools and training can minimize transmission risks. By fostering collaboration between traditional and formal healthcare systems, stakeholders can enhance disease prevention and control efforts, ultimately contributing to more resilient healthcare systems in Africa. This approach acknowledges the cultural significance of traditional healers and promotes a unified and culturally sensitive strategy to tackle the ongoing Mpox outbreak and other future public health outbreaks.

## Introduction

Mpox is a zoonotic viral disease endemic in Central and West Africa. The virus was first identified in 1958 [1], with the initial human case reported in 1970 involving a child in the Democratic Republic of Congo [2]. Since then, several countries have reported an Mpox outbreak, with the most recent outbreak, starting in May 2022, affecting hundreds of countries, including those that have never reported any typical case [3]. Since that time (May 2022), 85,189 Mpox cases have been reported from 110 countries globally (45,755 cases and 135 deaths from 30 WHO member states in Africa), prompting the World Health Organization (WHO) to declare it a public health emergency of international concern in July 2022 [3]. This outbreak has further weakened the healthcare system across many African countries, making healthcare service delivery extremely difficult, partly attributed to the prevention and control measures implemented to curtail its spread [4].

In the Democratic Republic of Congo (DRC), the strain in the healthcare system caused by Mpox has resulted in multiple outbreaks like cholera [5]. Factors like civil war, poverty, and recurring outbreaks like Marburg, Lassa fever, and Ebola have further exacerbated the impact of the ongoing Mpox outbreak in the African continent [6,7]. The ongoing conflict in DRC, for example, has posed a major challenge in the containment of the outbreak, which further intensifies the poverty rate of the population, as the majority cannot afford to work in the midst of the ongoing war [5]. These challenges have compounded and weakened the health system’s response to Mpox, which has implications for the attainment of the Sustainable Development Goals.

Between January 1, 2022 and September 28, 2025, a total of 54,906 Mpox cases and 239 deaths have been recorded from 30 WHO member states in Africa including Nigeria, Ghana, Sierra Leone, among others [8]. The Democratic Republic of Congo has been the epicenter of the outbreaks, with 21,772 cases [8]. Sierra Leone became the latest country in Africa to declare an Mpox outbreak on January 10, 2025 [9], three years after its global spread prompted the World Health Organization to declare it a Public Health Emergency of International Concern in May 2022 [10]. As of September 28, 2025, 5345 Mpox cases have been recorded in Sierra Leone [8]. Several reports have indicated traditional healers as the first point of contact for febrile and no-febrile illnesses in Africa, especially in rural areas [11–13].

Traditional healers in Africa are recognized for their role in treating a variety of common febrile illnesses, such as malaria, as well as mental health disorders, including depression and anxiety [14]. Factors contributing to the preference for traditional healing practices include relatively lower treatment costs, the accessibility of services within local communities, and the influence of system of thoughts (or superstitious belief) [15]. These elements collectively drive individuals to seek care from traditional healers.

This healthcare-seeking behavior is not exclusive to Mpox but a widespread practice among individuals with both febrile and non-febrile conditions across the African continent. For instance, a study conducted among Ebola survivors on the use of complementary and traditional medicine in Sierra Leone revealed that almost half of the survivors reported using traditional medicine [16]. Furthermore, a report by the World Health Organization highlights the critical role of traditional healers in several African countries [17]. In Sudan, the introduction of village midwives has significantly improved maternal healthcare service delivery [17]. Additionally, in Ghana and Nigeria, traditional healers have been shown to enhance primary healthcare services, particularly in rural areas, by facilitating community-based contraceptive delivery and promoting social well-being [17].

Despite the contributions of traditional healers in healthcare service delivery, especially in rural areas, there are concerns about how their services negate early treatment, which constitutes a huge public health risk. A study in Sierra Leone reported a delay in seeking healthcare by Lassa fever patients due to healthcare seeking from traditional healers [18]. This pattern of traditional medicine use during outbreaks underscores the need for these viewpoints. Barriers such as difficulties in accessing medical facilities, elevated healthcare expenses, poverty, the trust and familiarity of traditional healers within communities, and cultural and system of thought (philosophical beliefs of people deep-rooted from culture) impact the use of traditional medicine [19]. Although traditional healers play a pivotal role in healthcare service delivery in Africa [20], there is limited information on their practices and influence in managing outbreaks. This viewpoint investigated the role of traditional healers in Africa’s public health response with a focus on Mpox outbreaks and proposes strategies for collaboration. Specifically, it seeks to determine whether their practices act as a barrier to effective public health responses or if they can serve as a bridge to strengthen community-based disease prevention and control efforts. The medical pluralism/health-seeking behaviour theoretical framework was used to guide the arguments in analysing the coexistence of multiple healthcare systems (biomedical, traditional, spiritual) and how individuals and communities choose among them, particularly during a crisis like an Mpox outbreak.

## Role of traditional healers in treating infectious and non-infectious diseases with epidemic potential in Africa

Traditional healers play a crucial role in healthcare delivery across African countries in managing diseases with unknown origin by biomedical scientists, especially those associated with supernatural causes like mental disorders [21]. Their services are utilized by nearly 58.2% ranging between 4.6%-94% of the African population [21], reflecting their entrenched presence in cultural and traditional practices. Research indicates that traditional healers frequently serve as the initial contact for febrile illnesses in Africa, especially within rural communities [22]. In South Africa, a study among COVID-19 patients revealed that all participants reported receiving treatment from traditional healers, with none experiencing adverse effects post-treatment [23]. The study revealed the effectiveness and affordability of traditional medicine as key factors promoting its use, while barriers included a lack of knowledge and challenges in locating practitioners [23]. In North-western Nigeria, a preference for traditional medicine over conventional medicine was noted among respondents for treating loved ones infected with the Ebola virus [24]. Similarly, in Sierra Leone, nearly half of Ebola survivors engaged with traditional medicine [25]. Those who perceived traditional and complementary medicine as enhancing their immune system, having fewer side effects, and providing greater autonomy compared to conventional medicine were more inclined to use traditional practices [25]. This widespread utilization underscores the role of traditional medicine across the African continent. In the context of the ongoing Mpox outbreak, although research is limited, a study from Nigeria indicated that participants expressed a strong belief in the efficacy of traditional herbal remedies for treating Mpox cases. This reliance on traditional remedies has resulted in a reluctance to adopt conventional interventions, such as vaccines and other treatments [26]. The interconnectedness of traditional and conventional medicine highlights the need for a holistic approach to the treatment and management of cases during the current Mpox outbreak and in future public health emergencies. Traditional healers possess valuable local contextual knowledge of African settings, informed by cultural beliefs, and enriched by community trust and relationships. This unique position offers opportunities to enhance disease prevention and control, particularly during public health emergencies. The disconnect between traditional medicine and biomedical medicine arises from differing epistemologies, as modern medicine often adopts a reductionist approach focused on biological mechanisms, while traditional medicine emphasizes holistic perspectives that consider social, spiritual, and environmental factors.

Aside from emergency contexts, traditional healers offer treatment for a wide range of conditions. For instance, a study in Nigeria reported traditional healers offer treatment for severe malaria, especially those with convulsion history [27]. The authors also revealed that though traditional healers are crucial in treating malaria cases, the majority of whom do not make timely referral of cases to the health facility due to trust in the effectiveness of the efficacy of their herbal remedies [27]. These treatment practices cause delay in the treatment of cases in modern healthcare facilities, in most instances by two weeks [27]. Another study from Nigeria reported malaria as the most common illness treated by traditional healers. The authors reported boiled herbs, ground herbs, and incision and scarification as the methods used for delivering traditional healing procedures [28]. Further, the authors reported younger adult and those with at least secondary education were factors associated with likely referral of cases in modern healthcare facilities, underscoring the need for training, inclusion into the formal healthcare system, and continuous multisectoral, coordination and engagement as suggested practice to improve treatment of cases [28]. In Ghana, a report revealed traditional healers treat every illness including common cold, diabetes, hypertension, anxiety, depression, and infertility [29]. They also provide postnatal care services and serve as community mobilisers and advocates referring patients to modern healthcare facilities for surgeries and vaccination services and intensifying awareness raising initiatives like hygiene, nutrition, and disease prevention [29]. In Kenya, a study reported that traditional healers treat some childhood diseases like malaria, and a large proportion of the population believes they can treat measles, and instances where conventional medicines fail to provide diagnosis or is slow, traditional medicines are offering the last resort [30]. In South Africa, a study reported traditional healers are a crucial force in the treatment and management of mental disorders like anxiety and depression, substance use disorder, and mood disorder. The use of this service is almost at par with conventional medicines, with a mean visit of 2.7 (n = 57) for traditional healers versus 3.4 (n = 26) for conventional medicine in a year [31]. Furthermore, a study conducted in Ethiopia indicated that traditional healers provide treatment for a range of conditions, including cancer, wounds, fractures, paralysis, inflammation, herpes zoster, hemorrhoids, back pain, liver diseases, and eczema [32].

## Challenges posed by traditional healers in Mpox outbreak

Despite the critical role played by traditional healers in the management and treatment of diseases in Africa, their practices often do not align with modern medical standards, thereby undermining public health intervention efforts [33]. One of the primary reasons resulting to this misalignment is the non-integration of their practice into modern practice, which is guided by policies and regulations. This poses risks, including delays in seeking treatment at formal healthcare facilities, increased morbidity and mortality, and challenges to modern healthcare services. Traditional healers often hold considerable influence within their communities, which can be a double-edged sword. While they can serve as vital sources of support and guidance during health crises, their beliefs about the origins and treatment of diseases like Mpox may conflict with biomedical understandings. For instance, some traditional healers may view Mpox as a condition linked to spiritual or environmental factors rather than a viral infection, leading to alternative treatment approaches that may not be effective. It is crucial to acknowledge the substantial challenges arising from the differing conceptualizations of health and disease within biomedical and traditional frameworks. In rural communities, for instance, traditional healing practices exert considerable influence, as many individuals interpret illnesses through a lens that attributes their system of thoughts (origin to supernatural forces or devine intervention). This perspective often leads to a reliance on traditional herbal remedies, which are perceived as more cost-effective and are favored by low-income family members and influential community stakeholders. In contrast, urban populations frequently regard traditional medicine as antiquated, preferring modern biomedical approaches. These individuals tend to possess a broader understanding of the benefits associated with biomedical interventions and have greater access to such services and cost to afford them. This shift reflects a broader societal trend in which biomedical perspectives increasingly dominate health discourse, resulting in the marginalization of traditional healing practices. Given these challenges and the critical role of traditional healers, integrating them into outbreak response efforts is imperative to reduce disease transmission and fatalities. Strategies for achieving this include fostering collaborative partnerships between traditional healers and healthcare professionals, promoting mutual respect for different healing practices, and developing training programs that educate traditional healers about biomedical concepts while allowing them to share their knowledge. By bridging the gap between traditional and biomedical systems, we can enhance the overall response to health crises like Mpox.

## Delay in case detection and treatment

A major challenge identified in using traditional remedies during the ongoing Mpox outbreak is the delay in symptom recognition and timely referral to healthcare providers [34]. In Sierra Leone, for example, report indicated that the index case sought treatment from a traditional healer and this process lasted for several days, consequently resulting in the late diagnosis of the Mpox outbreak [35]. Despite several reports affirming the substantial roles played by traditional healers in disease identification [32,36], a report from Uganda also suggests traditional healers often lack the necessary knowledge to identify Mpox cases and other febrile illnesses. The authors further revealed that there is a lack of the required knowledge to refer patients presenting with Mpox symptoms to the nearest health facilities in a timely manner [37]. Consequently, this leads to delays in the identification of Mpox cases, thereby increasing the risk of disease transmission within the community and exacerbating the severity of the disease.

### Misinformation

Misinformation presents a critical challenge posed by traditional healers during public health emergencies in Africa [38]. Public health awareness, essential for dispelling rumors and misinformation, receives minimal funding from stakeholders during health crises. This results in a significant gap in public health knowledge regarding Mpox among the population. Traditional healers, whose practices are often motivated by profit, may attribute the symptoms of Mpox to spiritual causes or other non-scientific explanations, such as witchcraft [14]. Other factors like enhancing their social status in communities and a calling from God or a gift performed for humanity drive their motivation for practice. This perspective is deeply embedded in the cultural and social contexts of the communities they serve, where illness is viewed through system of thoughts (spiritual and supernatural lenses). Such beliefs contribute to a broader conceptualization of disease burden, encompassing not only physical symptoms but also social stigma and fear. The reliance on traditional healing practices reflects community trust in these systems, often arising from historical experiences with formal healthcare that may have been perceived as inadequate [39]. Consequently, the propagation of misinformation by traditional healers can hinder the acceptance of conventional medical interventions, including vaccinations.

## Non-adherence to infection, prevention, and control measures

Another critical challenge posed by traditional healers is their lack of adherence to infection prevention and control (IPC) measures, which significantly contributes to the spread of Mpox in the African region [40]. This lack of adherence is driven by system of thought (superstitious belief) that adherence to IPC protocols could lower the effectiveness of their treatment [41–43]. As Mpox is primarily transmitted through close contact with an infected individual or contaminated materials, the absence of proper IPC practices among traditional healers creates a high-risk environment for disease transmission. Many traditional healers operate without formal training or awareness of IPC protocols, such as the use of personal protective equipment (PPE), proper hand hygiene, safe handling of infectious materials, and disinfection of tools and surfaces [44]. This gap in knowledge and practice increases the risk of infection for patients and exposes the healers and their communities to the virus. By fostering collaboration between traditional and formal healthcare systems, it is possible to reduce the risk of Mpox transmission while respecting the cultural significance of traditional healing practices.

## Undermining public health interventions

As stakeholders in the African continent strive to halt the transmission of Mpox through initiatives such as contact tracing and advocating for vaccines to international partners [45], the exclusion of traditional healers from the public health response undermines these efforts [46]. Therefore, stakeholders must consider the integration of traditional healers as a crucial element in enhancing activities such as contact tracing, vaccine distribution, and community awareness. Addressing this gap necessitates recognizing the integration of traditional healers as an essential component of the Mpox response.

## Opportunities for collaboration in Africa’s public health response

The challenges posed by traditional healers also come with opportunity, as it enables the public health system to engage with them in improving healthcare delivery and in effectively responding to infectious disease outbreaks. The following strategies are proposed for the integration of traditional healers into the management of the Mpox outbreak and other future public health interventions:

### Training and education

The government, through the National Public Health Agency or Institutes (NPHA/I) in collaboration with the National Union of Traditional healers in most African countries including Nigeria [47], should conduct training for traditional healers on the early identification of Mpox symptoms and other febrile illnesses, and understand the referral protocol. This training should be done at the regional level, and traditional healers residing in rural settings should also be included. The NPHA/I should design a mentoring and supervision framework to check the activities of traditional healers for case referrals of Mpox symptoms or other febrile illnesses [48]. Over time, the NPHA/I should develop a standardised training framework for traditional healers, emphasising disease detection, prevention, and management.

### Community engagement

The NPHA/I, with support from partners should actively include traditional healers in public health campaigns and ensure their participation is incentivized [49,50]. To effectively enhance their capacity to participate in public health campaigns, stakeholders should develop tailored training programs for traditional healers. In the community, stakeholders should consider giving them leadership roles with supervision from trained health education experts to enhance community acceptance and adherence to prevention and control interventions of Mpox and other febrile illnesses. By integrating traditional healers into public health campaigns, incentivising their participation, and empowering them with leadership roles under expert supervision, stakeholders can bridge the gap between traditional and modern healthcare systems. While education is essential, a multifaceted approach is necessary for meaningful integration. This includes developing collaborative training programs that educate both traditional healers and healthcare professionals and engaging traditional healers in public health initiatives to leverage their influence within communities. Other interventions included: advocating for policies that formalize their role in healthcare and their limitations and conducting research to document their practices and effectiveness. Socio-anthropologists can contribute by conducting ethnographic studies to understand cultural beliefs surrounding health, facilitating dialogue between traditional healers and healthcare providers, and evaluating the impact of integrated approaches on community health outcomes [51]. Together, these strategies can foster a cohesive healthcare system that respects and incorporates both traditional and modern practices.

## Referral systems

Stakeholders through the NPHA/I should develop clear referral pathways for referral of suspected Mpox cases or other febrile illnesses to formal healthcare facilities [52]. This process must be designed to accommodate the unique challenges of rural and underserved areas, where traditional healers often serve as the first point of contact for healthcare. Staff from the NPHA/I should ensure that traditional healers are trained on the referral pathways and provide them with a standard operating procedure for the referral pathway document. Additionally, the NPHA/I should conduct periodic assessments to evaluate the utilisation and effectiveness of the referral pathways, ensuring they are achieving the desired outcomes in reducing delays in diagnosis and treatment [53]. This integration would enhance early detection and treatment of Mpox and other febrile illnesses, ultimately improving health outcomes and reducing the burden of disease in Africa.

## Infection control

Stakeholders at the NPHA/I should integrate traditional healers into the formal healthcare system and provide them with IPC tools like gloves, masks, and hand sanitizers [54,55]. Providing these tools is critical in ensuring traditional healers can safely interact with patients while minimising the risk of disease transmission, particularly for highly contagious illnesses like Mpox and other febrile conditions. Stakeholders should provide capacity-building training on using IPC materials and their importance in reducing Mpox transmission and other febrile illnesses [56]. Stakeholders through the NPHA/I should regularly conduct on-the-spot checks to ensure traditional healers adhere to IPC protocol [57]. This collaborative approach strengthens the overall public health response and fosters trust and cooperation between traditional healers and the formal healthcare sector, ultimately contributing to more effective disease prevention and control efforts.

## Conclusion

The Mpox outbreak in Africa shows the critical need to address the role of traditional healers in public health responses. While their practices pose challenges, their integration into formal healthcare systems offers an opportunity to strengthen outbreak management efforts. By fostering collaboration through training, community engagement, and establishing referral systems, traditional healers can serve as vital allies in reducing disease transmission and fatalities, ultimately contributing to more resilient healthcare systems across the African continent and other regions. This approach acknowledges the critical role of traditional healers in their communities while promoting a unified and culturally sensitive strategy to tackle future public health outbreaks.
